# Numerical Investigation of Darcy–Forchheimer Hybrid Nanofluid Flow with Energy Transfer over a Spinning Fluctuating Disk under the Influence of Chemical Reaction and Heat Source

**DOI:** 10.3390/mi14010048

**Published:** 2022-12-25

**Authors:** Muhammad Riaz Khan, Aisha M. Alqahtani, Sharifah E. Alhazmi, Mohamed Abdelghany Elkotb, Maawiya Ould Sidi, Haifaa F. Alrihieli, Elsayed Tag-Eldin, Mansour F. Yassen

**Affiliations:** 1 Department of Mathematics, Quaid-i-Azam University, Islamabad 44000, Pakistan; 2 Department of Mathematical Sciences, College of Science, Princess Nourah bint Abdulrahman University, Riyadh 11671, Saudi Arabia; alqahtani@pnu.edu.sa; 3 Mathematics Department, Al-Qunfudah University College, Umm Al-Qura University, Mecca 21955, Saudi Arabia; sehazmi@uqu.edu.sa; 4 Mechanical Engineering Department, College of Engineering, King Khalid University, Abha 61421, Saudi Arabia; melkotb@kku.edu.sa; 5 Mechanical Engineering Department, College of Engineering, Kafrelsheikh University, Kafr Elsheikh 33516, Egypt; 6 RT-M2A Laboratory, Mathematics Department, College of Science, Jouf University, P.O. Box 2014, Sakaka 42421, Saudi Arabia; msidi@ju.edu.sa; 7 Department of Mathematics, Faculty of Science, University of Tabuk, P.O. Box 741, Tabuk 71491, Saudi Arabia; halreheili@ut.edu.sa; 8 Faculty of Engineering and Technology, Future University in Egypt, New Cairo 11835, Egypt; elsayed.tageldin@fue.edu.eg; 9 Department of Mathematics, College of Science and Humanities in Al-Aflaj, Prince Sattam Bin Abdulaziz University, Al-Aflaj 11912, Saudi Arabia; mf.ali@psau.edu.sa; 10 Department of Mathematics, Faculty of Science, Damietta University, New Damietta 34517, Egypt

**Keywords:** wavy fluctuating disk, hybrid nanofluid, MHD, PCM, chemical reaction, heat source

## Abstract

The present computational model is built to analyze the energy and mass transition rate through a copper and cobalt ferrite water-based hybrid nanofluid (hnf) flow caused by the fluctuating wavy spinning disk. Cobalt ferrite (CoFe_2_O_4_) and copper (Cu) nanoparticles (nps) are incredibly renowned in engineering and technological research due to their vast potential applications in nano/microscale structures, devices, materials, and systems related to micro- and nanotechnology. The flow mechanism has been formulated in the form of a nonlinear set of PDEs. That set of PDEs has been further reduced to the system of ODEs through resemblance replacements and computationally solved through the parametric continuation method. The outcomes are verified with the Matlab program bvp4c, for accuracy purposes. The statistical outputs and graphical evaluation of physical factors versus velocity, energy, and mass outlines are given through tables and figures. The configuration of a circulating disk affects the energy transformation and velocity distribution desirably. In comparison to a uniform interface, the uneven spinning surface augments energy communication by up to 15%. The addition of nanostructured materials (cobalt ferrite and copper) dramatically improves the solvent physiochemical characteristics. Furthermore, the upward and downward oscillation of the rotating disc also enhances the velocity and energy distribution.

## 1. Introduction

The study of the hybrid nanofluid (hnf) flow over a spinning disc with energy and mass transitions has a significant commitment to current innovations and advanced applications. Some of them are electric power generation systems, biomedical devices, aeronautical science, co-rotating apparatus, rotating devices, chemical reactions, the hydrothermal sector, and optical computing [1,2,3,4]. For a Darcy–Forchheimer hybrid ferrofluid flow across a permeable whirling disc, Li et al. [5] developed fractional and computational models using iron oxide nanoparticulates. Bilal et al. [6] analyzed the implications of a zigzag intermittent revolving disc with energy transmission on a ferric oxide and carbon nanotubes water-based hybrid NF flow. The buildup of biocomposites was thought to considerably improve the liquid medium’s thermal properties. Zhang et al. [7] documented the 3D computational formulation of the MgO-Ag/water hnf flow with momentum and heat transfer produced by an irregular moving disk. The shape of a turning disc has been discovered to have a promising upshot on speed and energy conversion. Khan [8] addressed the influence of entropy production in a semi-radiation flow of hybrid NPs in a viscous fluid over a turning disc. Waqas et al. [9] employed the bvp4c code to describe the process of the radiative hnf flow across a spinning disc with activating energy and floating microbes. Zhou et al. [10] addressed the Maxwell hnf flow across a whirling disc with the suction and injection mechanism. The mass conveyance seems to increase when the thermophoresis factor is increased, whereas the velocity decreases as the viscosity parameter is improved. Using the PCM methodology, Lv et al. [11] documented the upshot of magnetic flux, Hall current, and heat radiation on a hnf flow composed of CNTs and magnetic nps across the edge of a rotating disc. Sadiq et al. [12] documented a customized lubrication to support that the dependability, risk-free operation, and stability of various bearings is accelerated by advances in the mechanical understanding and the strict demand for rotating systems of heavy machinery. Further applications and studies of fluid flow over a gyrating disk can be found in some recent literature [13,14,15,16].

Hybrid nanofluid is a novel class of fluid, which is useful in the energy transition. Hybrid nanoliquids may be employed to thermal activities, such as freezing, solar energy, heat pumps, heat convertors, air conditioners, transmitters, motorized sectors, electric coolers, radioactive systems, ships, and biosciences [17,18]. When copper is introduced to freshwater for varnishes, polymers, and textiles, it serves as an antibiotic, antifungal, and antimicrobial agent. Copper dietary supplements have a high rate of absorption. Copper has high-tensile-strength metals and alloys [19,20,21]. Metallic cobalt Co and iron Fe ions have a significant role in the elevation of heat capacitance [22]. Imaging processing is new and advances the application of nanofluid. The most flexible among them is MRI, which can offer both functional and morphological information while maintaining outstanding picture quality. Bi-magnetic particles are employed to make them more functional. Cobalt ferrite NPs with bi-magnetic core–shell features have appeared as a viable alternative for developing a novel MRI contrast. Bi-magnetic nps may also be employed for medication delivery and are an ideal candidate for developing new nano-theragnostic medicines [23].

The mathematical approach to the above-mentioned applications and problems are discussed by several mathematicians and researchers. Among them, Alharbi et al. [24,25] inspected the numerical study of nanofluid over a stretching sheet and wedge. Ramesh et al. [26] added CoFe_2_O_4_ and Fe_3_O_4_ nano-mixtures in water + EG to execute the covalent bonding. Wang et al. [27] dispersed MWCNT and Fe_3_O_4_ nanomaterials in hybrid nanoliquid to simulate the thermal efficiency of a traditional solid heat sink. Salahuddin et al. [28] explored the 3D peristaltic flow of a hnf as it flows through an extensible heated wavy cylinder with varying thickness and slips circumstances. Ibrahim et al. [29] numerically considered the influence of twisted turbulators on improving energy proficiency through a hnf for a solar collector. Wang et al. [30] offered guidelines about how to build and produce nanoscale granules by trifunctional materials. The FeZn_4_Co/CNFs electrocatalyst was found to be one of the effective compounds for energy communication. Ullah et al. [31] mathematically analyzed the effects of entropy in the Darcy–Forchheimer stream of hybrid nanostructures made of CNTs and kerosene oil (base fluid). Skin friction was thought to increase with inertia coefficients, porosity factor, and the rotation constraint. Nazeer et al. [32] calculated the hnf flow through a micro-channel using electro-osmatic nanocomposites. The computed finding showed that the velocity contour decreased concerning the electro-kinetic variable, magnetic field factor, and viscosity component. Chu et al. [33] considered the magnetism and bioconvection effect on the Maxwell hnf on an extending cylinder. It was found that the percentage of microbes decreases as the quantities of the Peclet number increase. Some related literature and applications of CoFe_2_O_4_ and Cu nps in the water for biomedical and engineering purposes may be initiated in [34,35].

The purpose of this study is to expand an idea suggested by Mohebbi et al. [36], by studying the consequence of the different nanoparticles, Cu and CoFe_2_O_4_ water-based hybrid NFs, on a wavy circling fluctuating disc. The second priority is to augment the productivity and implementation of thermal energy conveyance for a range of biological, industrial, and commercial uses. In order to maximize the thermal efficiency of water-based hybrid nanoliquid across a rotating surface, this paper investigates the effects of a nano composition and MHD on the hnf flow. The Darcy–Forchhemier, chemical reaction, and heat source terms all contributed to the study’s uniqueness.

## 2. Governing Equations

**Figure 1:**
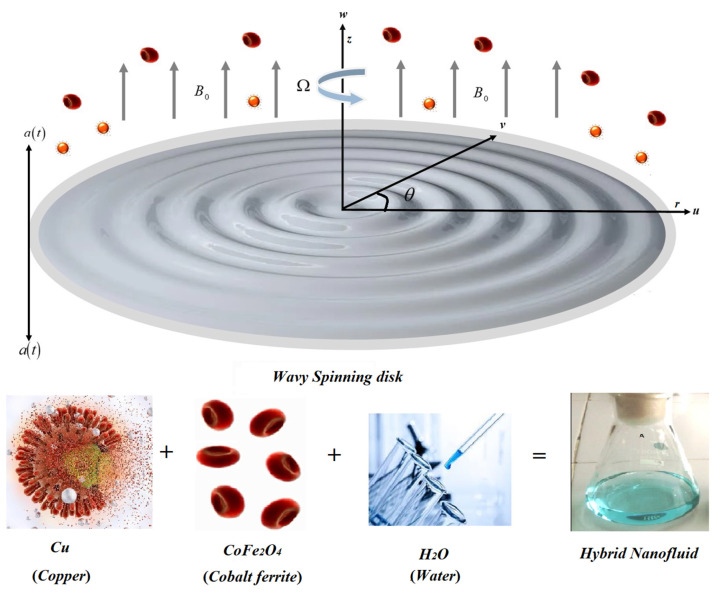
The hybrid nanofluid flow over a fluctuating disk.

We assumed a 3D unsteady hybrid NF flow comprised of Cu and CoFe_2_O_4_ nano particulates over a fluctuating wavy moving gyrating disc. Initially, the disc is at $a\left(0\right)=h$ . Then, with some movement $\omega =a\left(t\right)$ (angular velocity), the disc moves at $Z=a\left(t\right)$ in the vertical direction. The disc moves with the velocity $\Omega \left(t\right)$ at the z-axis as shown in Figure 1. The buoyancy effect is presumed to be neglected. It is supposed that the Cu and CoFe_2_O_4_ nanoparticulate nanomaterials are disseminated homogenously. The buoyant impacts are minimal, proving that they are insignificant when compared to the flow’s inertia force. The magnetic effect is employed uniformly. On behalf of exceeding presumptions, the basic equations are expressed as [37,38,39]:

(1) $$\frac{\partial u}{\partial r}+\frac{\partial w}{\partial z}+\frac{u}{r}=0,$$

(2) $$\rho_{hnf}\left(\frac{\partial u}{\partial t}+u\frac{\partial u}{\partial r}+w\frac{\partial u}{\partial z}-\frac{v^{2}}{r}\right)=-\frac{\partial p}{\partial r}+\mu_{hnf}\left(\frac{\partial^{2}u}{\partial r^{2}}+\frac{\partial^{2}u}{\partial z^{2}}-\frac{u}{r^{2}}+\frac{1}{r}\frac{\partial u}{\partial r}\right)-\frac{\nu }{k^{*}}u-Fu^{2}+F_{r},$$

(3) $$\rho_{hnf}\left(\frac{\partial v}{\partial t}+u\frac{\partial v}{\partial r}+w\frac{\partial v}{\partial z}-\frac{uv}{r}\right)=\mu_{hnf}\left(\frac{\partial^{2}v}{\partial r^{2}}+\frac{\partial^{2}v}{\partial z^{2}}-\frac{v}{r^{2}}+\frac{1}{r}\frac{\partial v}{\partial r}\right)-\frac{\nu }{k^{*}}v-Fv^{2},$$

(4) $$\rho_{hnf}\left(\frac{\partial w}{\partial t}+u\frac{\partial w}{\partial r}+w\frac{\partial w}{\partial z}\right)=-\frac{\partial p}{\partial z}+\mu_{hnf}\left(\frac{\partial^{2}w}{\partial r^{2}}+\frac{\partial^{2}w}{\partial z^{2}}+\frac{1}{r}\frac{\partial w}{\partial r}\right)-\frac{\nu }{k^{*}}w-Fw^{2}+F_{\theta },$$

(5) $$\left(\frac{\partial T}{\partial t}+u\frac{\partial T}{\partial r}+w\frac{\partial T}{\partial z}\right)=\frac{k_{hnf}}{{\left(\rho C_{p}\right)}_{hnf}}\left(\frac{\partial^{2}T}{\partial r^{2}}+\frac{1}{r}\frac{\partial T}{\partial r}+\frac{\partial^{2}T}{\partial z^{2}}\right)+\frac{Q_{0}\left(T-T_{\infty }\right)}{\rho C_{p}},$$

(6) $$\left(\frac{\partial C}{\partial t}+u\frac{\partial C}{\partial r}+w\frac{\partial C}{\partial z}\right)=D_{hnf}\left(\frac{\partial^{2}C}{\partial r^{2}}+\frac{\partial^{2}C}{\partial z^{2}}+\frac{1}{r}\frac{\partial C}{\partial r}\right)-Kr\left(C-C_{\infty }\right),$$

Here, $F_{r}$ and $F_{\theta }$ are the body forces along *x* and *z* directions defined as [37]:

(7) $$F_{r}=\frac{Ha^{2}\mu_{hnf}}{R^{2}}\left(v\sin\theta \cos\theta -u\sin^{2}\theta \right),\;\;\;F_{\theta }=\frac{Ha^{2}\mu_{hnf}}{R^{2}}\left(u\sin\theta \cos\theta -v\sin^{2}\theta \right).$$

Here, *Ha* is the Hartmann number $\left(Ha=LB_{0}\sqrt{\frac{\sigma }{\mu }}\right)$ and θ is the direction, whereas, in the above equations, *Kr*, *k*, and *Q*_0_ are the chemical reaction rate, porosity term, and heat source, respectively.

The associated boundary conditions are:

(8) $$\left.\begin{array}{l} u=0,\;\;\;w=\beta \overset{*}{a}\left(t\right),\;\;v=r\Omega_{0}\left(t\right),\;\;C=C_{0},\;\;\;\;T=T_{0}\;\;\;\mathrm{at}\;\;\;\;z=0\\ u\to 0,\;\;w\to 0,\;\;v\to 0,\;\;\;C\to C_{\infty },\;\;\;\;T\to T_{\infty }\;\;\mathrm{at}\;\;\;z\to \infty . \end{array}\right\}$$

The transformation variables are:

(9) $$\left.\begin{array}{l} u=\frac{rv}{a^{2}\left(t\right)}f\left(\eta \right),\;\;w=\frac{v}{a\left(t\right)}h\left(\eta \right),\;\;v=\frac{rv}{a^{2}\left(t\right)}g\left(\eta \right),\;\;\;p=\frac{pv^{2}}{a^{2}\left(t\right)}p\left(\eta \right),\;\;C=C_{\infty }+\Delta C\Phi ,\\ \;\eta =\frac{Z}{a\left(t\right)}-1,\;\;\;T=T_{\infty }+\Delta T\theta ,\;\;\eta_{Z}=\frac{1}{a\left(t\right)},\;\eta_{t}=\frac{-a\left(t\right)}{a\left(t\right)}\left(\eta +1\right). \end{array}\right\}$$

By incorporating Equation (9), we obtain:

(10) $$f^{\prime \prime }=\frac{\rho_{hnf}}{\mu_{hnf}}\left(hf^{\prime }+f^{2}-g^{2}-S\frac{\left(\eta +1\right)f^{\prime }}{2}+f-\lambda f^{\prime }-Fr{f^{\prime }}^{2}\right)+A\omega \left(g\cos\theta \sin\theta -f\sin^{2}\theta \right),$$

(11) $$g^{\prime \prime }=\frac{\rho_{hnf}}{\mu_{hnf}}\left(hg^{\prime }+2fg-S\left(\frac{\left(\eta +1\right)g^{\prime }}{2}-g-\lambda g-Frg^{2}\right)\right),$$

(12) $$h^{\prime \prime }=\frac{\rho_{hnf}}{\mu_{hnf}}\left(hh^{\prime }-S\frac{\left(\eta +1\right)h^{\prime }}{2}+h^{\prime }-\lambda h-Frh^{2}\right)-A\omega \left(f\sin\theta \cos\theta -g\sin^{2}\theta \right),$$

(13) $$\theta {\left(\eta \right)}^{\prime \prime }=\rho_{hnf}\left(Prh\theta {\left(\eta \right)}^{\prime }-PrS\left(\frac{\left(\eta +1\right)\theta {\left(\eta \right)}^{\prime }}{2}+\gamma \theta \left(\eta \right)\right)\right)+\hbar \theta \left(\eta \right),$$

(14) $$\Phi^{\prime \prime }=Sc\;h\Phi^{\prime }-Sc\;S\left(\frac{\eta +1}{2}\right)\Phi^{\prime }+Kr\Phi ,$$

The transform conditions are:

(15) $$\left.\begin{array}{l} f\left(0\right)=0,\;\;\;h\left(0\right)=\beta \frac{S}{2},\;\;\;\theta \left(\eta \right)\left(0\right)=1,\;\;g\left(0\right)=\omega ,\;\;\;\Phi \left(0\right)=1\;\;\;\mathrm{at}\;\;\eta \to 0,\\ f\left(\eta \right)\to 0,\;g\left(\eta \right)\to 0,\;h\left(\eta \right)\to 0,\;\theta \left(\eta \right)\left(\eta \right)\to 0,\;\;\Phi \left(\eta \right)\to 0\;\;\;\;\;\mathrm{as}\;\eta \to \infty . \end{array}\right\}$$

Here, *S* is the disk fluctuation term, *Kr* is the rate of chemical reaction, ω is the disk’s rotation, λ is the porosity parameter, *Fr* is the Forchheimer factor, γ is the thermal energy ratio constraint, and ℏ is the heat source defined as:

(16) $$\begin{array}{l} S=2\frac{a^{*}\left(t\right)a\left(t\right)}{v},\;\;Kr=\frac{K_{c}a^{2}\left(t\right)}{v_{f}},\;\;\omega =2\frac{a^{2}\left(t\right)\Omega \left(t\right)}{v},\;\;\lambda =\frac{\nu }{k^{*}\Omega },\;\;Fr=\frac{C_{b}}{k^{*1/2}},\\ \gamma =\frac{1}{2}\frac{a\left(t\right)T}{a^{*}\left(t\right)\Delta T},\;\;\hbar =\frac{xQ_{0}}{\rho C_{p}}. \end{array}$$

The physical quantities are:

(17) $$C_{f}=\frac{\sqrt{\tau_{wr}^{2}+\tau_{w\phi }^{2}}}{{\left(\Omega r\right)}^{2}\rho_{f}},\;\;\;Nu=\frac{q_{w}\;r}{\left(T_{w}-T_{\infty }\right)k_{f}},\;\;\;\;Sh=\frac{j_{w}\;r}{\left(C_{w}-C_{\infty }\right)D_{f}}.$$

where

(18) $$\tau_{wr}={\left[\left(\frac{du}{dz}+\frac{dw}{d\phi }\right)\left(\mu_{hnf}\right)\right]}_{z=0},\tau_{w\phi }={\left[\left(\frac{dv}{dz}+\frac{1}{r}\frac{dw}{d\phi }\right)\left(\mu_{hnf}\right)\right]}_{z=0},q_{w}=-\left(\frac{k_{hnf}}{k_{f}}\right){\left(\frac{dT}{dz}\right)}_{z=0},j_{w}=-\left(D_{hnf}\right){\left(\frac{dC}{dz}\right)}_{z=0}.$$

The dimensionless form of Equation (17) is:

(19) $$Re^{\frac{1}{2}}C_{f}=\frac{\sqrt{{\left(G^{\prime }(0)\right)}^{2}+{\left(F^{\prime }(0)\right)}^{2}}}{{\left(1-\phi_{1}\right)}^{2.5}{\left(1-\phi_{2}\right)}^{2.5}},\;\;Re^{\frac{-1}{2}}Nu=-\frac{k_{hnf}}{k_{f}}\theta^{\prime }\left(\eta \right)(0),\;Re^{\frac{-1}{2}}Sh=-\Phi^{\prime }(0),\;\;Re=\frac{\Omega r^{2}}{\upsilon_{f}}.$$

## 3. Numerical Solution

The basic procedure of the PCM approach applied to a set of ODEs (Equations (10)–(15)) is functionalized as [40,41]:

**Step 1:** Simplifying Equations (10)–(14) to 1st order

(20) $$\left.\begin{array}{l} \delta_{1}(\eta )=f(\eta ),\;\;\;\delta_{2}=f^{\prime }(\eta ),\;\;\;\delta_{3}=g(\eta ),\;\;\;\;\delta_{4}=g^{\prime }(\eta ),\;\;\;\;\delta_{5}=h(\eta ),\;\;\;\;\;\;\delta_{6}=h^{\prime }(\eta ),\;\;\;\delta_{7}(\eta )=\theta (\eta ),\;\;\\ \delta_{8}=\theta^{\prime }(\eta ),\;\;\;\delta_{9}=\Phi (\eta ),\;\;\;\delta_{10}=\Phi^{\prime }(\eta ). \end{array}\right\}$$

By putting Equation (20) in Equations (10)–(15), we obtain:

(21) $${\delta_{2}}^{\prime }=\frac{\rho_{hnf}}{\mu_{hnf}}\left(\left(\delta_{5}-S\frac{\left(\eta +1\right)}{2}\right)\delta_{2}+{\left(\delta_{1}\right)}^{2}-{\left(\delta_{3}\right)}^{2}-S\delta_{1}-\lambda \delta_{1}-Fr{\delta_{1}}^{2}\right)+A\omega \left(\delta_{3}\sin\theta \cos\theta -\delta_{1}\sin^{2}\theta \right),$$

(22) $${\delta_{4}}^{\prime }=\frac{\rho_{hnf}}{\mu_{hnf}}\left(\left(\delta_{5}-S\frac{\left(\eta +1\right)}{2}\right)\delta_{4}+2\delta_{1}\delta_{3}-S\delta_{3}-\lambda \delta_{3}-Fr{\delta_{3}}^{2}\right),$$

(23) $${\delta_{6}}^{\prime }=\frac{\rho_{hnf}}{\mu_{hnf}}\left(\left(\delta_{5}-S\frac{\left(\eta +1\right)}{2}+1\right)\delta_{6}-\lambda \delta_{5}-Fr{\delta_{5}}^{2}\right)-A\omega \left(\delta_{1}\sin\theta \cos\theta -\delta_{3}\sin^{2}\theta \right)$$

(24) $${\delta_{8}}^{\prime }=\rho_{hnf}\left(\left(Pr\delta_{5}-PrS\frac{\left(\eta +1\right)}{2}\right)\delta_{8}-S\delta_{7}\gamma \right)+\hbar \delta_{7},$$

(25) $${\delta^{\prime }}_{10}=Sc\;S\delta_{10}-Sc\;S\left(\frac{\left(\eta +1\right)\delta_{10}}{2}+\lambda \delta_{7}\right)+Kr\delta_{9},$$

The transform conditions are:

(26) $$\begin{array}{l} \delta_{1}=0,\;\;\;\;\delta_{3}=\omega ,\;\;\;\;\delta_{5}=\beta \frac{S}{2},\;\;\;\;\delta_{7}=1,\;\;\;\;\delta_{9}=1\;\;\;\;\mathrm{at}\;\;\;\;\;\eta \to 0,\\ \delta_{1}\to 0,\;\;\;\;\delta_{3}\to 0,\;\;\;\delta_{5}\to 0,\;\;\;\delta_{7}\to 0,\;\;\;\delta_{9}\to 0\;\;\;\mathrm{at}\;\;\;\;\eta \to \infty . \end{array}$$

**Step 2:** Introducing parameter *p*:

(27) $${\delta_{2}}^{\prime }=\frac{\rho_{hnf}}{\mu_{hnf}}\left(\left(\delta_{5}-S\frac{\left(\eta +1\right)}{2}\right)\left(\delta_{2}-1\right)p+{\left(\delta_{1}\right)}^{2}-{\left(\delta_{3}\right)}^{2}-S\delta_{1}-\lambda \delta_{1}-Fr{\delta_{1}}^{2}\right)+A\omega \left(\begin{array}{l} \delta_{3}\sin\theta \cos\theta \\ -\delta_{1}\sin^{2}\theta \end{array}\right),$$

(28) $${\delta_{4}}^{\prime }=\frac{\rho_{hnf}}{\mu_{hnf}}\left(\left(\delta_{5}-S\frac{\left(\eta +1\right)}{2}\right)\left(\delta_{4}-1\right)p+2\delta_{1}\delta_{3}-S\delta_{3}-\lambda \delta_{3}-Fr{\delta_{3}}^{2}\right),$$

(29) $${\delta_{6}}^{\prime }=\frac{\rho_{hnf}}{\mu_{hnf}}\left(\left(\delta_{5}-S\frac{\left(\eta +1\right)}{2}+1\right)\left(\delta_{6}-1\right)p-\lambda \delta_{5}-Fr{\delta_{5}}^{2}\right)-A\omega \left(\delta_{1}\sin\theta \cos\theta -\delta_{3}\sin^{2}\theta \right),$$

(30) $${\delta_{8}}^{\prime }=\rho_{hnf}\left(\left(Pr\delta_{5}-PrS\frac{\left(\eta +1\right)}{2}\right)\left(\delta_{8}-1\right)p-S\delta_{7}\gamma \right)+\hbar \delta_{7},$$

(31) $${\delta^{\prime }}_{10}=Sc\;S\left(\delta_{10}-1\right)p-Sc\;S\left(\frac{\left(\eta +1\right)\delta_{10}}{2}+\lambda \delta_{7}\right)+Kr\delta_{9},$$

## 4. Results and Discussion

**Figure 2:**
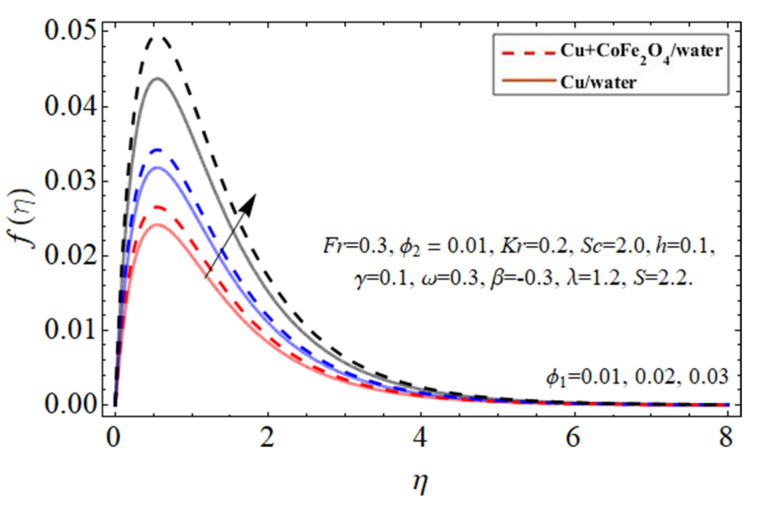
The behavior of primary velocity $f\left(\eta \right)$ against copper $\phi_{1}=\phi_{Cu}$ nanoparticles.

**Figure 3:**
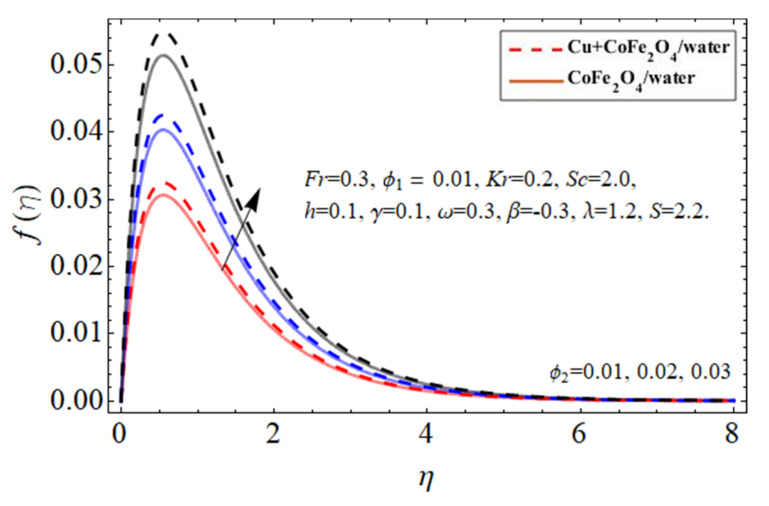
The behavior of primary velocity $f\left(\eta \right)$ against cobalt ferrite $\phi_{2}=\phi_{Fe_{2}O_{4}}$ nanoparticles.

**Figure 4:**
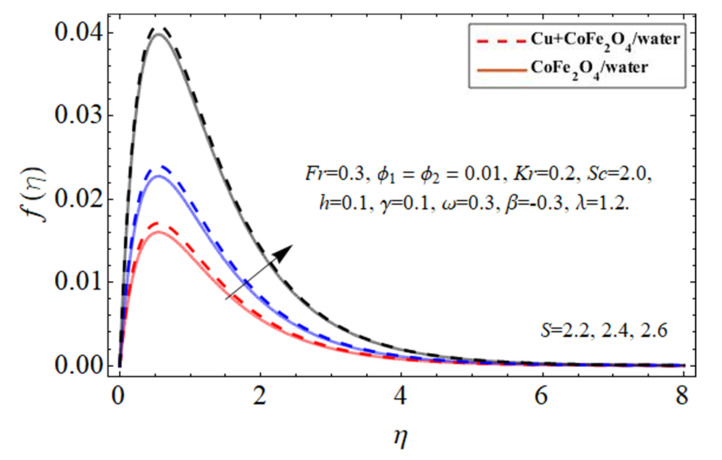
The behavior of primary velocity $f\left(\eta \right)$ against disk fluctuation term *S*.

**Figure 5:**
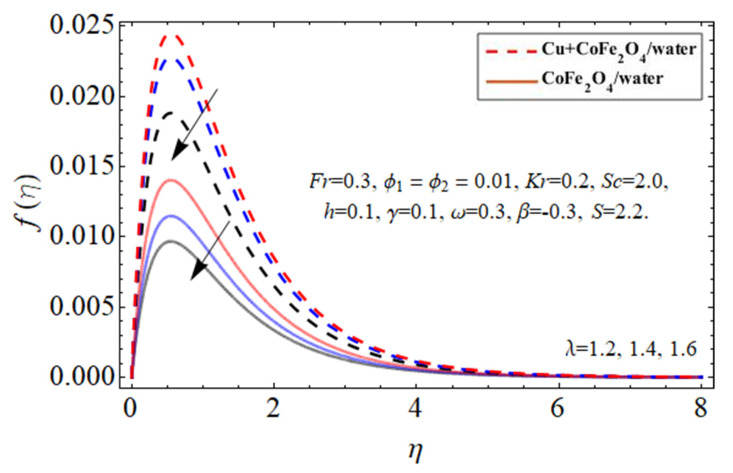
The behavior of primary velocity $f\left(\eta \right)$ against porosity parameter λ .

**Figure 6:**
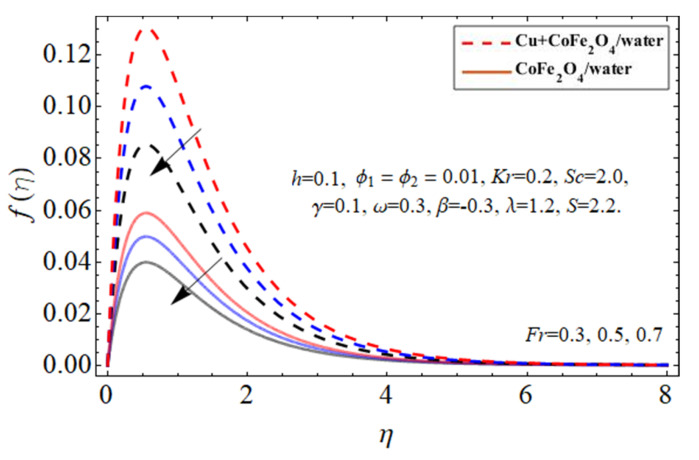
The behavior of primary velocity $f\left(\eta \right)$ against Forchhemier number *Fr*.

This section reveals the physical trend and explains the mechanism behind each result. The following observations have been made:

Figure 2, Figure 3, Figure 4, Figure 5 and Figure 6 explain the outlines of the velocity $f\left(\eta \right)$ field against the variation in $\phi_{1}=\phi_{Cu}$ , cobalt ferrite $\phi_{2}=\phi_{Fe_{2}O_{4}}$ , disk fluctuation parameter *S*, porosity term λ , and Forchhemier number *Fr*, respectively. Because water’s specific heat ability is more than those of the Cu and cobalt ferrite nanostructures, including them in the base fluid decreases their average heat absorption efficiency, leading to a rise in fluid acceleration as illustrated in Figure 2 and Figure 3. The upward and downward oscillation of the turning disc encourages molecules of water to transfer instantly, raising the fluid’s axial velocity as perceived in Figure 4. It is obvious that the porosity and Forchhemier number lessen the fluid velocity as reported in Figure 5 and Figure 6. The variation in porosity term λ enhances the fluid kinetic viscosity while declining the disk rotation rate, so as a result, the flow speed diminishes.

**Figure 7:**
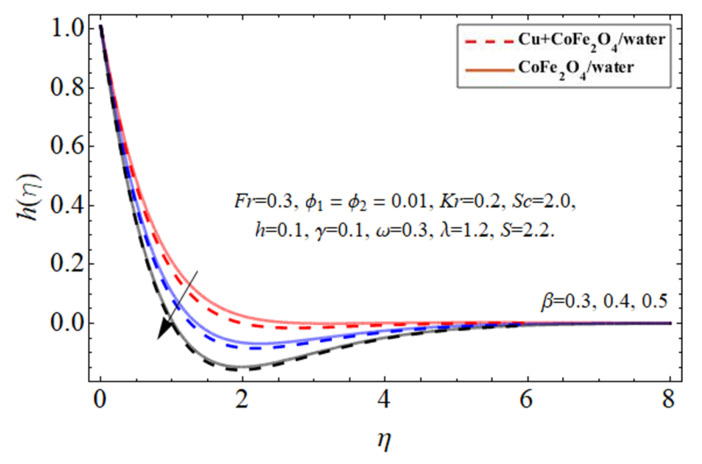
The behavior of secondary velocity $h\left(\eta \right)$ against injection term +β .

**Figure 8:**
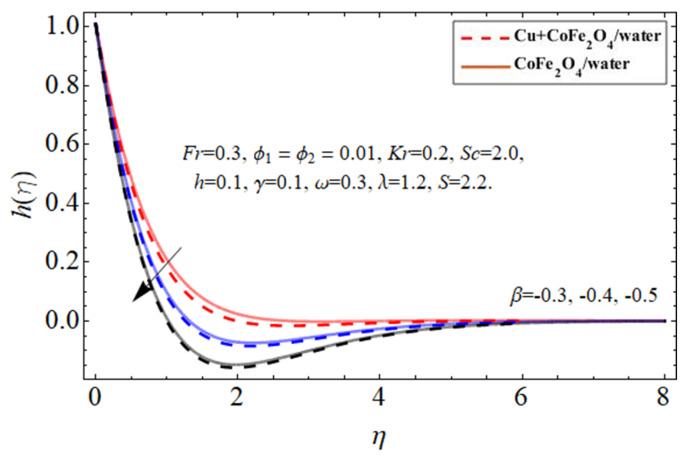
The behavior of secondary velocity $h\left(\eta \right)$ against suction term −β .

**Figure 9:**
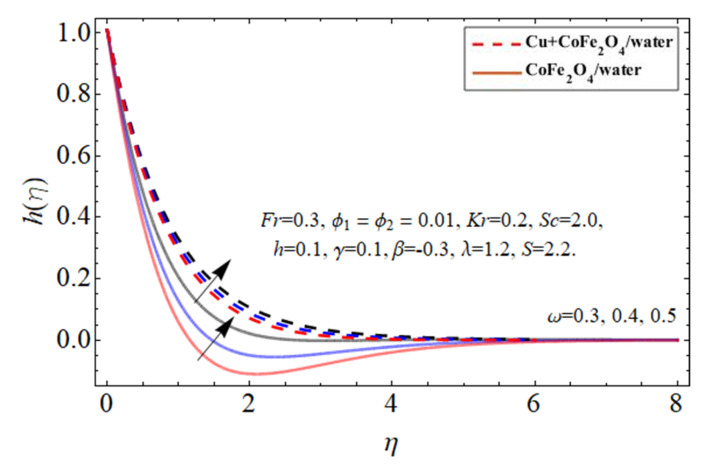
The behavior of secondary velocity $h\left(\eta \right)$ against disk term ω .

Figure 7, Figure 8 and Figure 9 particularize the radial velocity $h\left(\eta \right)$ profile trend against the injection +β term, suction −β coefficient, and disk spinning constant ω influence, respectively. Both sucking and infusion effects on the edge of the revolving disc provide an impedance to the flow stream, resulting in a drop in the peripheral flow velocity, as seen in Figure 7 and Figure 8. The increasing disc centrifugal acceleration also energizes the fluid particulates, causing an intensification in fluid radial velocity across an irregular surface as highlighted in Figure 9.

**Figure 10:**
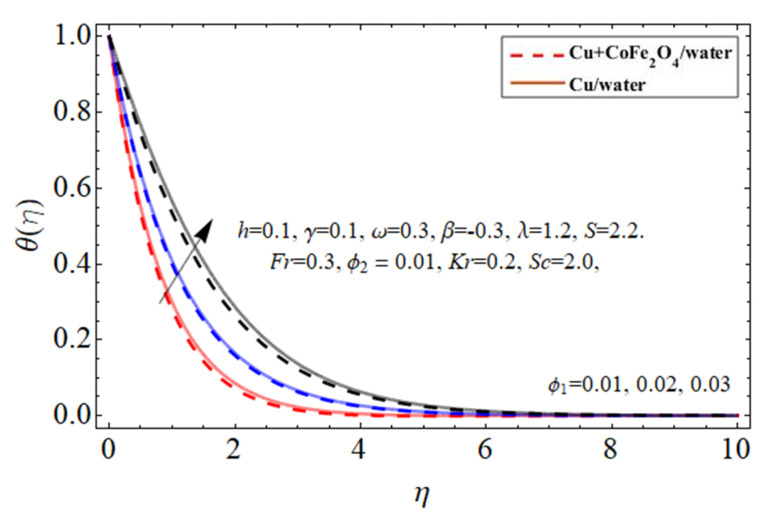
The nature of energy $\theta \left(\eta \right)$ field against copper $\phi_{1}$ nanoparticles.

**Figure 11:**
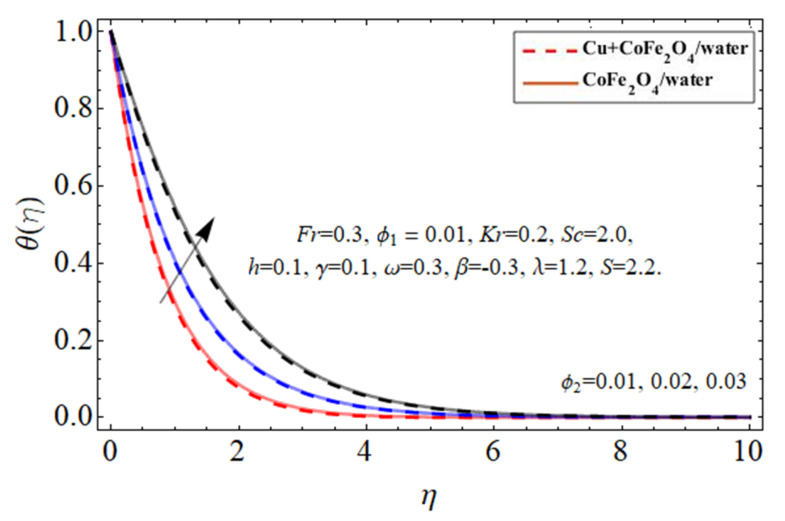
The nature of energy $\theta \left(\eta \right)$ field against cobalt ferrite $\phi_{2}$ nanoparticles.

**Figure 12:**
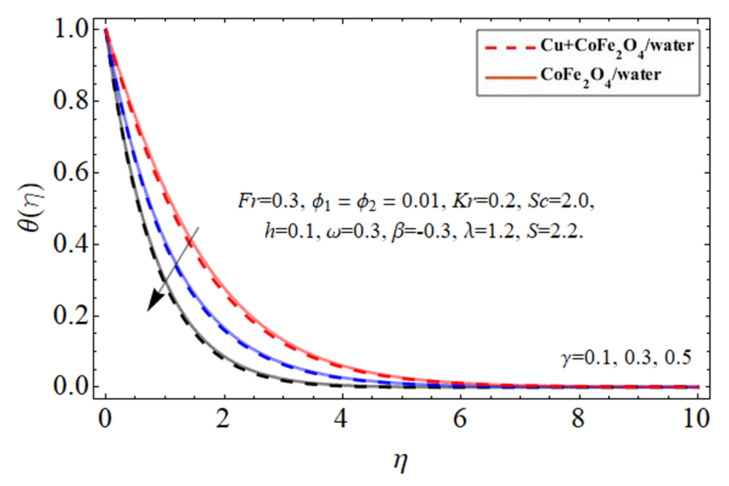
The nature of energy $\theta \left(\eta \right)$ field against the thermal energy ratio coefficient γ .

**Figure 13:**
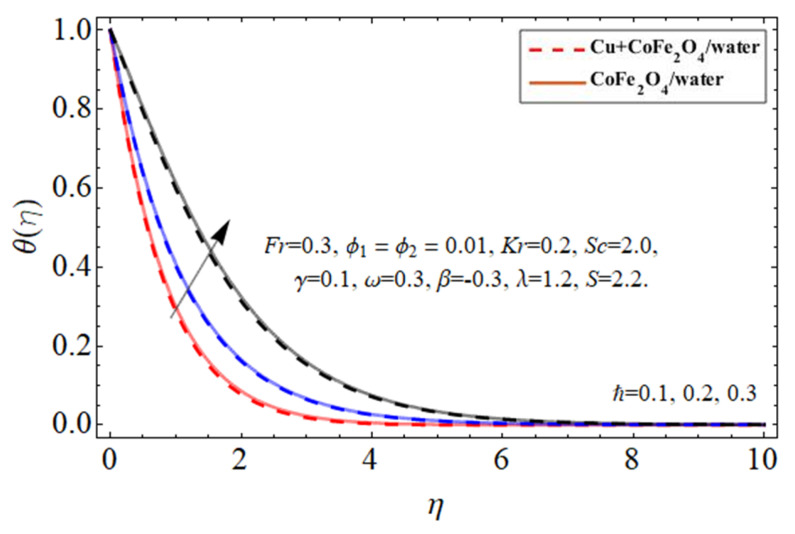
The nature of energy $\theta \left(\eta \right)$ versus heat absorption/generation term ℏ .

Figure 10, Figure 11, Figure 12 and Figure 13 demonstrate the behavior of the heat $\theta \left(\eta \right)$ profile via the copper Cu $\phi_{1}$ nanomaterial, cobalt ferrite $\phi_{2}$ nanoparticles, thermal energy ratio term γ , and heat source ℏ , correspondingly. Because water’s specific heat ability is more than those of the Cu and cobalt ferrite nanostructures, dispersing such nanostructures in a working fluid decreases its heat flux absorbency, increasing the fluid temperature as seen in Figure 10 and Figure 11. This property of nano particulates in the hybrid nanofluid makes it more valuable for the biomedical and engineering field because their inclusion improves the thermal efficiency of base fluid, which is mostly used in medical and industrial apparatus. As shown in Figure 12 and Figure 13 the thermal energy conveyance rate decreases when the thermal power ratio component γ improves, whereas it tends to increase as the heat absorption/generation term rises.

**Figure 14:**
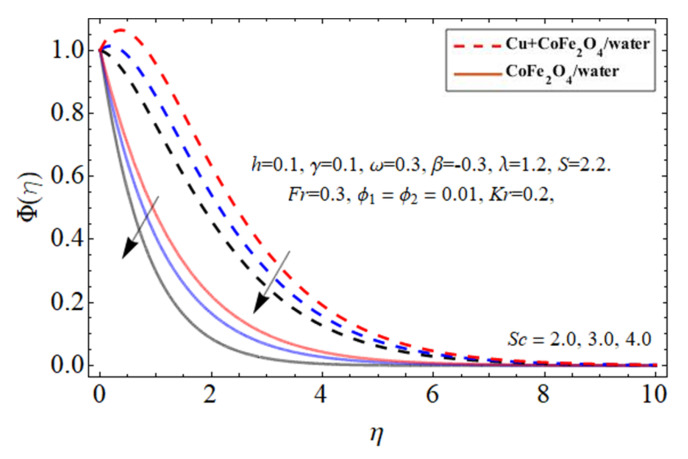
The nature of concentration $\Phi \left(\eta \right)$ profile versus the Schmidt number *Sc*.

**Figure 15:**
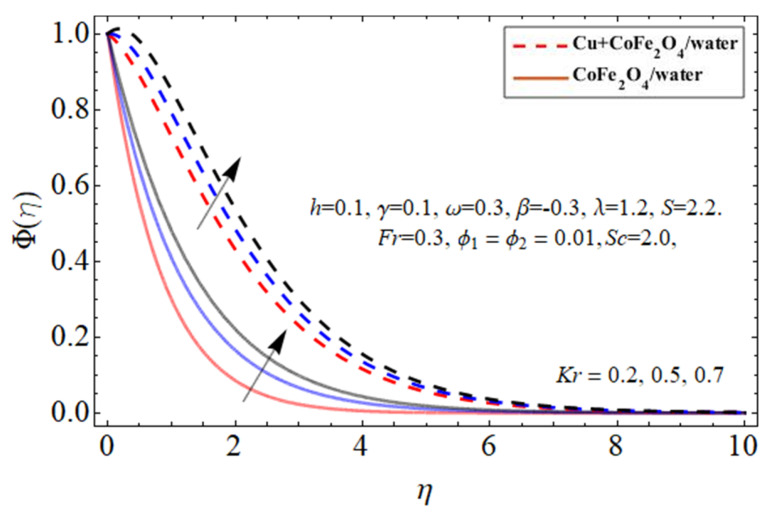
The nature of concentration $\Phi \left(\eta \right)$ profile versus the chemical reaction *Kr*.

Figure 14 and Figure 15 display the performance of the mass transference $\Phi \left(\eta \right)$ profile versus *Sc* and *Kr*, respectively. As revealed in Figure 14, the Sc affects the mass passing rate of hybrid nanofluids. The action of Sc enhances the kinetic viscosity of a viscous fluid flow while decreasing the molecular dissolution rate, resulting in the rehabilitation of mass transmission. On the other hand, the mass conversion ratio boosts with the increase in *Kr*.

Table 1 and Table 2 represent the thermal properties and experimental values of CoFe_2_O_4_ and Cu nano particulates, respectively. Table 3 describes the numerical valuation of the bvp4c and published work with the PCM results, to ensure accuracy. The velocity profiles and energy fields are associated with the determination. Table 4 and Table 5 establish the relative scrutiny for the Nusselt number and skin friction amid copper and copper ferrite hybrid nanoliquid.

## 5. Conclusions

The computational estimation of hybrid nanoliquid comprised of CoFe_2_O_4_ and Cu nanomaterial flows caused by the oscillation of a rotating wavy disc with energy dissemination is described in the proposed investigation. The goal of the suggested study is to advance the reliability of thermal energy transportation for a diversity of commercial and biological sectors. The observations are described as a system of PDEs that are graphically and statistically calculated using the PCM procedure. Below are the main discoveries from the aforesaid assessment:

- The dispersion of copper Cu $\left(\phi_{1}=\phi_{Cu}\right)$ and cobalt ferrite $\left(\phi_{2}=\phi_{CoFe_{2}O_{4}}\right)$ nanoparticles in the working fluid water significantly boosts the mass and energy transfer rate.
- The upward and downward oscillation of the turning disc encourages molecules of water to transfer instantly, raising the fluid’s axial velocity.
- The variation in porosity term λ and Forchhemier number *Fr* reduces the fluid velocity.
- Both sucking −β and infusion +β effects on the texture of the revolving disc provide an impedance to the flow stream, which drops the fluid velocity.
- The effect of thermal energy ratio term γ reduces, while the heat source ℏ term improves the fluid temperature.
- The mass transference $\Phi \left(\eta \right)$ profile falls with the outcome of *Sc* and boosts with the *Kr* factor.

## Figures and Tables

**Table 1:** The experimental values of water and nano particulates [38].

	$\rho (kg/m^{3})$	$C_{p}(j/kgK)$	k(W/mK)
Water	997.1	4179	0.613
Copper Cu	8933	385	401
CoFe_2_O_4_	4907	700	3.7

**Table 2:** The thermal characteristics of the hybrid nanoliquid $\left(\phi_{1}=\phi_{Cu},\;\phi_{2}=\phi_{CoFe_{2}O_{4}}\right)$ [39].

Properties	
Viscosity	$\frac{\mu_{hnf}}{\mu_{bf}}=\frac{1}{{\left(1-\phi_{Cu}-\phi_{CoFe_{2}O_{4}}\right)}^{2}}$
Density	$\frac{\rho_{hnf}}{\rho_{bf}}=\phi_{Cu}\left(\frac{\rho_{Cu}}{\rho_{bf}}\right)+\phi_{CoFe_{2}O_{4}}\left(\frac{\rho_{CoFe_{2}O_{4}}}{\rho_{bf}}\right)+\left(1-\phi_{Cu}-\phi_{CoFe_{2}O_{4}}\right)$
Thermal Capacity	$\frac{{\left(\rho C_{p}\right)}_{hnf}}{{\left(\rho C_{p}\right)}_{bf}}=\phi_{Cu}\left(\frac{{\left(\rho C_{p}\right)}_{Cu}}{{\left(\rho C_{p}\right)}_{bf}}\right)+\phi_{CoFe_{2}O_{4}}\left(\frac{{\left(\rho C_{p}\right)}_{CoFe_{2}O_{4}}}{{\left(\rho C_{p}\right)}_{bf}}\right)+\left(1-\phi_{Cu}-\phi_{CoFe_{2}O_{4}}\right)$
Thermal Conductivity	$\frac{k_{hnf}}{k_{bf}}=\left[\frac{\left(\frac{\phi_{Cu}k_{Cu}+\phi_{CoFe_{2}O_{4}}k_{CoFe_{2}O_{4}}}{\phi_{Cu}+\phi_{CoFe_{2}O_{4}}}\right)+2k_{bf}+2\left(\phi_{Cu}k_{Cu}+\phi_{CoFe_{2}O_{4}}k_{CoFe_{2}O_{4}}\right)-2\left(\phi_{Cu}+\phi_{CoFe_{2}O_{4}}\right)k_{bf}}{\left(\frac{\phi_{Cu}k_{Cu}+\phi_{CoFe_{2}O_{4}}k_{CoFe_{2}O_{4}}}{\phi_{Cu}+\phi_{CoFe_{2}O_{4}}}\right)+2k_{bf}-2\left(k_{Cu}\phi_{Cu}+k_{CoFe_{2}O_{4}}\phi_{CoFe_{2}O_{4}}\right)+\left(\phi_{Cu}+\phi_{CoFe_{2}O_{4}}\right)2k_{bf}}\right]$
Electrical Conductivity	$\frac{\sigma_{hnf}}{\sigma_{bf}}=\left[\frac{\left(\frac{\phi_{Cu}\sigma_{Cu}+\sigma_{CoFe_{2}O_{4}\;}\phi_{CoFe_{2}O_{4}}}{\phi_{CoFe_{2}O_{4}}+\phi_{Cu}}\right)+2\sigma_{bf}+2\left(\phi_{Cu}\sigma_{Cu}+\phi_{CoFe_{2}O_{4}}\sigma_{CoFe_{2}O_{4}}\right)-2\left(\phi_{Cu}+\phi_{CoFe_{2}O_{4}}\right)\sigma_{bf}}{\left(\frac{\phi_{Cu}\sigma_{Cu}+\phi_{CoFe_{2}O_{4}}\sigma_{CoFe_{2}O_{4}}}{\phi_{Cu}+\phi_{CoFe_{2}O_{4}}}\right)+2\sigma_{bf}-\left(\phi_{Cu}\sigma_{Cu}+\phi_{CoFe_{2}O_{4}}\sigma_{CoFe_{2}O_{4}}\right)+\left(\phi_{Cu}+\phi_{CoFe_{2}O_{4}}\right)\sigma_{bf}}\right]$

**Table 3:** Comparative assessments of the present work with the existing study and bvp4c method.

	Zhang et al. [7]			Present Results (PCM)			Present Results (bvp4c)		
η	$f\left(\eta \right)$	$g\left(\eta \right)$	$\theta \left(\eta \right)$	$f\left(\eta \right)$	$g\left(\eta \right)$	$\theta \left(\eta \right)$	$f\left(\eta \right)$	$g\left(\eta \right)$	$\theta \left(\eta \right)$
0.0	0.0000	0.0000	1.0000	0.0000	0.0000	1.0000	0.0000	0.0000	1.0000
0.1	0.0002	0.0020	0.2711	0.0035	0.0027	0.2719	0.0031	0.0021	0.2321
0.3	0.0060	0.0157	0.0572	0.0067	0.0177	0.0582	0.00060	0.0172	0.0173
0.5	−0.0391	−0.0771	0.0093	−0.0399	−0.0788	0.0073	−0.0353	−0.1332	0.0061
0.7	−0.1359	−0.1913	0.0019	−0.1369	−0.1923	0.0023	−0.1320	−0.2103	0.0017

**Table 4:** The relative assessment of Cu and cobalt ferrite nanoparticulate on skin friction.

	Cu		CoFe_2_O_4_	
η	$f^{\prime }\left(0\right)$	$g^{\prime }\left(0\right)$	$f^{\prime }\left(0\right)$	$g^{\prime }\left(0\right)$
0.00	0.2723	1.4911	0.3321	0.5561
0.05	0.4132	1.5723	0.7733	0.6820
0.01	0.6640	1.7053	1.0010	0.7921
0.15	1.0301	2.0790	1.1700	0.1900
0.20	1.1531	2.2752	1.3531	1.3101

**Table 5:** Statistical assessments of Sherwood and Nusselt numbers.

	Cu		CoFe_2_O_4_	
η	$\Theta^{\prime }\left(0\right)$	$\Phi^{\prime }\left(0\right)$	$\Theta^{\prime }\left(0\right)$	$\Phi^{\prime }\left(0\right)$
0.00	1.4954	1.6954	1.4581	1.6934
0.03	1.2362	1.5362	1.5732	1.5315
0.06	1.2482	1.3482	1.3621	1.3417
0.09	1.0014	1.1012	2.1683	1.1032

## Data Availability

The data that support the findings of this study are available within the article.
